# The prevalence of neutropenia and association with infections in patients with systemic lupus erythematosus: a Swedish single-center study conducted over 14 years

**DOI:** 10.1007/s00296-024-05566-9

**Published:** 2024-03-19

**Authors:** Muna Saleh, Johanna Sjöwall, Marcus Bendtsen, Christopher Sjöwall

**Affiliations:** 1 Department of Biomedical and Clinical Sciences, Division of Inflammation and Infection/Rheumatology, Linköping University, Linköping, Sweden; 2 Department of Biomedical and Clinical Sciences, Division of Inflammation and Infection/Infectious Diseases, Linköping University, Linköping, Sweden; 3 Department of Health, Medicine and Caring Sciences, Division of Society and Health, Linköping University, 581 83 Linköping, Sweden; 4 Rheumatology Unit, Linköping University Hospital, 581 85 Linköping, Sweden

**Keywords:** Systemic lupus erythematosus, Infection, Neutropenia, Lymphopenia, Hypocomplementemia

## Abstract

Hematologic abnormalities are common manifestations of SLE, although neutropenia is observed less frequently and is not included in the classification criteria. Nonetheless, neutropenia is a risk factor for infections, especially those caused by bacteria or fungi. We aimed to evaluate the impact of neutropenia in SLE through a systematic investigation of all infections in a large cohort of well-characterized patients, focusing on neutropenia, lymphopenia, and hypocomplementemia. Longitudinal clinical and laboratory parameters obtained at visits to the Rheumatology Unit, Linköping University Hospital, and linked data on all forms of healthcare utilization for all the subjects included in our regional SLE register during 2008–2022 were assessed. Data regarding confirmed infections were retrieved from the medical records. Overall, 333 patients were included and monitored during 3,088 visits to a rheumatologist during the study period. In total, 918 infections were identified, and 94 occasions of neutropenia (ANC < 1.5 × 10^9^/L) were detected in 40 subjects (12%). Thirty neutropenic episodes in 15 patients occurred in association with infections, of which 13 (43%) required in-hospital care, 4 (13%) needed intensive care, and 1 (3%) resulted in death. Bayesian analysis showed that patients with ≥ 1 occasion of neutropenia were more likely to experience one or more infections (OR = 2.05; probability of association [POA] = 96%). Both invasiveness (OR = 7.08; POA = 98%) and severity (OR = 2.85; POA = 96%) of the infections were significantly associated with the present neutropenia. Infections are common among Swedish SLE patients, 12% of whom show neutropenia over time. Importantly, neutropenia is linked to both the invasiveness and severity of infections. Awareness of the risks of severe infections in neutropenic patients is crucial to tailor therapies to prevent severe illness and death.

**Supplementary Information:**

The online version contains supplementary material available at 10.1007/s00296-024-05566-9.

## Introduction

The clinical spectrum of systemic lupus erythematosus (SLE) is highly heterogeneous, ranging from mild disease, which can be limited to skin and joint involvement, to life-threatening conditions that may involve renal impairment, severe cytopenia, central nervous system disease, and thromboembolic events [1]. Thus, SLE continues to represent a major challenge for both patients and physicians. Inappropriate or incomplete management of SLE disease activity and/or the side effects of administered therapies, particularly corticosteroids, may lead to comorbidities, irreversible organ damage, decreased health-related quality of life, and increased mortality [2–4].

Infections remain one of the leading causes of mortality and morbidity for patients with SLE [5]. The increased susceptibility to infections is related to the disease itself, as well as to the administered medications. SLE is associated with an increased risk of infections due to altered host immune status, i.e., hypocomplementemia, as well as due to abnormal neutrophil and macrophage responses to pathogens. In addition, immunosuppressive drugs, such as corticosteroids, cytotoxic agents and biologics, may affect the lymphocyte count as well as B and T cell functions and thereby impair the immune response to viral and bacterial pathogens, further contributing to the risk of infections [5–8].

Data from the USA, the UK, and Singapore indicate that approximately 50% of patients with SLE experience at least one severe infection during their disease course, and that 11%–23% of all hospitalizations among individuals with SLE are due to infections [9–11]. A recent study conducted by Simard et al*.* based on Swedish registry data found that individuals with incident SLE were two to four times more likely to be hospitalized for infections and experienced more recurrent infections than the general population. Among immunosuppressive agents, azathioprine was associated with the highest rate of infections [12]. Another European registry study has shown that bacterial infections account for 52% of all infections in patients with SLE, followed by viruses (12%) and fungi (2%) [13]. Regarding sites of infections, the respiratory tract (35%), urinary tract (15%), and soft tissues (13%) have been reported among individuals with SLE [13, 14]. However, although many bacterial infections are evidently more prevalent in patients with SLE than in the general population, the causal pathogens do not seem to differ much and include mainly *Staphylococcus aureus*, *Streptococcus pneumonia,* and *Escherichia coli* [5]. However, the rates of opportunistic infections may be underestimated in patients with SLE due to their similarities with disease flares [15, 16].

Hematologic abnormalities occur frequently in patients with SLE and are included in the classification criteria [17, 18]. Lymphopenia is particularly common and is associated with an increased risk of damage accrual [19]. However, cytopenias can have several etiologies, including disease activity, bone marrow failure, drug toxicity, peripheral cell destruction, tumor infiltration, and sepsis [20, 21]. Neutropenia is found less often than other cytopenias in patients with SLE [22]. The putative mechanism behind autoimmune neutropenia involves increased peripheral cell destruction by circulating anti-neutrophil antibodies [23], increased margination, changes in the marginal zone splenic cell pools [24], and decreased granulopoiesis, but other mechanisms may also be of relevance, e.g., drugs and infections [25, 26]. Most episodes of neutropenia in SLE are mild, with an absolute neutrophil count (ANC; < 1.5‒ ≥ 1.0 × 10^9^/L), although approximately 5% of patients may experience moderate (ANC < 1.0‒ ≥ 0.5 × 10^9^/L) to severe (ANC < 0.5‒ ≥ 0.2 × 10^9^/L) neutropenia [25]. Low serum levels of soluble Fc-γ receptor and high levels of granulocyte colony-stimulating factor have been proposed as risk factors for the development of infections in neutropenic patients with SLE [27, 28]. Furthermore, neutrophil function has also been demonstrated to be of high relevance in SLE [29].

The aims of this study were to; (i) identify systematically and characterize, over a period of 14 years, all the infections that occurred in a large cohort of Swedish patients with confirmed SLE; and (ii) explore any differences in infections that occurred in conjunction with or in the absence of neutropenia in these patients with SLE.

## Patients and methods

### Subjects

The patients in this retrospective observational study originated from the regional research program *Clinical Lupus Register in Northeastern Gothia* (Swedish acronym: *KLURING*) and fulfilled the 1982 American College of Rheumatology (ACR) and/or the 2012 Systemic Lupus International Collaborating Clinics (SLICC) classification criteria [17, 18]. This single-center cohort has recently been described in detail and includes all adult individuals (≥ 18 years of age) with SLE residing in Östergötland County; patients were continuously enrolled as prevalent or incident cases and longitudinally followed since 2008 at the Rheumatology Unit, Linköping University Hospital [30]. The current study encompassed all the included subjects followed prospectively until death, April 30th 2022 or emigration from the study region. Data on all healthcare consumption in the study region were available to us. At all visits to the rheumatologist, ongoing medications were registered, and disease activity was assessed using the clinical SLE disease activity index 2000 (SLEDAI-2 K) with exclusion of items for anti-dsDNA binding and hypocomplementemia. Blood samples for blood cell counts and assays for creatine kinase and complement protein (C)3 and C4, as well as urinalysis were monitored at each visit to the rheumatologist [30].

### Identification of neutropenia

During the healthcare visits, episodes of neutropenia, defined as ANC < 1.5 × 10^9^/L, were identified. This cutoff has been applied in previous studies of adults [31, 32]. Furthermore, with regard to the definitions derived from previous studies, neutropenia was here categorized as: mild, for ANC < 1.5‒ ≥ 1.0 × 10^9^/L; moderate, for ANC < 1.0‒ ≥ 0.5 × 10^9^/L; severe, for ANC < 0.5‒ ≥ 0.2 × 10^9^/L; and agranulocytosis, for ANC < 0.2 × 10^9^/L. In addition, the episodes of neutropenia were classified as either ‘occasional’ or ‘recurrent’. When one or more tests displayed neutropenia within a single 4-week time span, the neutropenia was considered to be occasional. If the neutropenia reoccurred over several 4-week time spans (at least two episodes), the neutropenia was considered to be recurrent.

### Identification and validation of infections

We conducted a systematic case record review of each included patient from the date of inclusion in *KLURING* until death, April 30th 2022, or emigration from the study region. The review was initially performed by M.S., consultant rheumatologist, and then further reviewed by J.S., senior consultant in infectious diseases. The collected data concerned the public healthcare utilization in the study region of all the included patients, reflecting infection-related complaints or symptoms (from either physical in-person or digital contacts with healthcare professionals). We included only those infections where the diagnosis was made by a physician based on typical symptoms, physical examinations, and laboratory, culture, or radiologic findings, and the diagnosis was required to have been documented, given a relevant ICD-10 code, and treated as an infectious disease. Data on the use of antibiotics as well as antiviral and antifungal drugs were available, including both prescriptions and agents provided in-hospital care.

### Characterization of infections

The identified infections were assigned to the following categories: (i) bacterial, viral, fungal, or mixed infections; (ii) affected organ system or localizations: bone, skin, and soft tissues, and respiratory, urogenital, gastrointestinal, neurologic, or multiple organ systems, in addition to infections with unspecified localizations; (iii) invasive and non-invasive infections (when the microorganism was found in the blood or in the cerebrospinal fluid); and (iv) the severity of the infections, depending on how they were treated and managed. Infection severity was rated on four levels: (1) infections treated in the outpatient care setting; (2) infections requiring in-hospital care; (3) infections necessitating intensive care; (4) and infections causing death.

The identified infections were divided into three subgroups: (a) infections that occurred in conjunction with confirmed neutropenia; (b) infections that occurred in the absence of neutropenia; and (c) infections in which neutropenia had not been verified with a white blood cell (WBC) differentiation test 2 months prior to or after the infectious episode.

For all neutropenic episodes, regardless of concomitant infection, information regarding hypocomplementemia and lymphopenia was available and neutrophil-to-lymphocyte ratio (NLR) could be provided. Hypocomplementemia was defined as C3 ≤ 0.69 g/mL and/or C4 ≤ 0.12 g/mL, according to the local laboratory at Linköping University Hospital, and lymphopenia was defined as a lymphocyte count < 1.1 × 10^9^/L. To increase the statistical power, fulfillment of the SLICC classification criteria for ‘lymphopenia’ and ‘low complement’ was used instead of concomitant hypocomplementemia and lymphocyte count in specified analyses [17].

### Statistics

To characterize the infections identified in the case review, we report descriptive statistics for infection type, location, invasiveness, and severity. Logistic regression analysis was used to estimate the odds of infection based on ever having experienced neutropenia, and for estimating the odds of infection when neutropenic episodes occurred in patients with ongoing or a history of hypocomplementemia and lymphopenia. For the models of co-occurrence, we added an adaptive intercept to the models, to account for the clustering of infections within patients. We also analytically estimated the associations between infection invasiveness and severity with co-occurring neutropenia, adjusted for whether patients had ever experienced hypocomplementemia and lymphopenia during the study period. In these models, we also added an interaction term between hypocomplementemia and lymphopenia (according to the SLICC criteria) [17]. We used ordinal regression for severity and logistic regression for invasiveness (infections for which no culture had been assessed were removed), with adaptive intercepts introduced to account for the clustering of infections within patients.

All the regression models were estimated using Bayesian inference [33]. We used Student *t*-distributions as priors for the intercepts and coefficients (degrees of freedom = 3, center = 0, scale = 2.5). We present the medians of the posterior distributions as point estimates, along with the 95% compatibility intervals (CI), defined by the 2.5% and 97.5% percentiles of the posterior distributions. The posterior probability of association (POA) in the direction of the median is also presented. All of the statistical analyses we performed with the R ver. 4.0.5 and Stan (CmdStan ver. 2.30.1) software packages [34, 35].

### Patient and public involvement

Patients or the public were not involved in the design, conduct, reporting, or dissemination plans of our research.

## Results

In total, 333 patients who were diagnosed with SLE based on the 1982 ACR and/or the 2012 SLICC classification criteria were included in the analyses [17, 18]. The majority of the cases were women (86%) and more than 88% of the patients were of Caucasian ethnicity/White race. Altogether, during the study period (2008‒2022), the patients were monitored at 3,088 visits to the rheumatologist. The characteristics of the included patients are listed in Table 1.

**Table 1:** Characteristics of the included patients

Items	Subjects (*n* = 333)
Background variables	
Age at SLE diagnosis, years, median (IQR)	34 (26.2–40.7)
Female gender, *n* (%)	284 (86)
Caucasian ethnicity/White race, *n* (%)	294 (88)
Clinical SLEDAI-2 K score, median (IQR)	0 (0–1)
Daily glucocorticoid use, number (%) of visits when any dose of glucocorticoid was prescribed	1993 (65)
Fulfilled classification criteria at last follow-up (ACR-82 definitions)	
Number of criteria fulfilled *n* (range)	4.8 (3–9)
1. Malar rash, *n* (%)	125 (38)
2. Discoid rash, *n* (%)	51 (15)
3. Photosensitivity, *n* (%)	173 (52)
4. Oral ulcers, *n* (%)	39 (12)
5. Arthritis, *n* (%)	255 (77)
6. Serositis, *n* (%)	118 (36)
7. Renal disorder, *n* (%)	92 (28)
8. Neurologic disorder, *n* (%)	20 (6)
9. Hematologic disorder, *n* (%)	206 (62)
10. Immunologic disorder, *n* (%)	184 (55)
11. IF-ANA, *n* (%)	328 (99)

*ACR-82* the 1982 American College of Rheumatology classification criteria, *IF-ANA* antinuclear antibodies analyzed with immunofluorescence microscopy, *IQR* interquartile range, *SD* standard deviation, *SLE* systemic lupus erythematosus, *SLEDAI-2 K* SLE Disease Activity Index 2000

### Episodes of neutropenia

We identified 94 episodes of neutropenia in 40 patients (12% of all included cases). Among these patients, the proportion of women was 90%. The average age at which neutropenic episodes occurred was 44.4 years (SD, 13). In 88/94 (94%) of the neutropenic episodes, combined ongoing hematologic manifestations (58 leukopenia, 41 lymphopenia, 36 anemia and 16 thrombocytopenia) were observed. Thus, only six episodes of isolated neutropenia were found. The mean NLR of the 94 neutropenic episodes was 1.03 (SD, 0.65). The most commonly detected autoantibody specificity in patients with neutropenic episodes was anti-SSA (Ro60 and/or Ro52/TRIM21), which was found in 62% of the neutropenic patients (42% of the overall study population). Details concerning the characteristics of the patients with episodes of neutropenia and ongoing treatments during neutropenic episodes are provided in Table 2.

**Table 2:** Type of neutropenic episodes and associations between confirmed neutropenia and disease activity, fulfilled classification criteria, presence of autoantibodies, and ongoing treatment

	Episodes (*n* = 94)	Percent of all episodes (%)	Patients (*n* = 40)
Occasional neutropenia (1 episode only)	24	26	24
Recurrent neutropenia (> 1 episode)	70	75	16
Mild neutropenia	62	66	36
Moderate neutropenia	21	22	8
Severe neutropenia	9	9.6	1
Agranulocytosis	2	2.1	2
Neutropenia with clinical SLEDA-2 K score ≤ 4	76	81	33
Neutropenia with ≥ 4 ACR criteria fulfilled	84	89	38
Neutropenia with Renal disorder (7th ACR criterion fulfilled)	16	17	12
Neutropenia with ≥ 1 concomitant hematologic manifestation	88	94	34
Neutropenia with positivity for anti-SSA antibodies (Ro60, Ro52/TRIM21)	58	62	27
Neutropenia with positivity for anti-SSB antibodies (La)	26	28	14
Neutropenia with Immunologic disorder (10th ACR criterion fulfilled)	39	42	25
Neutropenic episodes with ongoing daily treatment with 0–5 mg prednisolone	58	62	29
Neutropenic episodes with ongoing daily treatment with > 5 mg prednisolone	36	38	15
Neutropenic episodes with ongoing treatment with ≥ 1 immunosuppressive agent*	31	33	18
Neutropenic episodes with ongoing treatment with rituximab	8	9	6
Neutropenic episodes with ongoing treatment with belimumab	1	1	1

*Immunosuppressive agents provided: sirolimus (*n* = 11), cyclosporine (*n* = 5), methotrexate (*n* = 5), azathioprine (*n* = 4), mycophenolate mofetil (*n* = 4), leflunomide (*n* = 2), dapsone (n = 1), and cyclophosphamide (*n* = 1)

### Infections in patients with or without neutropenia

During the study period, a total of 918 infections was identified among 237 patients (71%). Regarding those patients who had at least one infection, 85% were women. The average age of the patients who experienced infections was 56.2 years (SD, 18.2).

Among the 918 infections, 30 (3.3%) co-occurred with confirmed neutropenia, clustered among 15 patients. Details regarding ongoing immunosuppressive therapy, daily glucocorticoid dose, NLR and the infecting pathogens are provided in Supplementary Table 1. The NLR among patients with higher severity of infection (level 3 or 4; mean 0.95, SD, 0.67) was not significantly different than in those with less severity of infection (level 1 or 2; mean 0.74, SD, 0.57). Both patients with agranulocytosis (Supplementary Table 1) received granulocyte–macrophage colony-stimulating factor (GM-CSF).

The remaining 888 infections could be divided into 274 infections (in 131 patients) without neutropenia and 614 infections (in 211 patients) in which WBC differentiation had not been assessed in conjunction with the infections (note that infections may have occurred more than once per patient, regardless of the ANC). Throughout the study period, the odds of infections were higher among individuals who (on any occasion) had experienced neutropenia than among those who had no confirmed neutropenic episodes (OR = 2.09, 95% CI  0.94;5.19, POA = 96.3%). The characteristics of the infections are presented in Table 3, divided according to the three neutropenia-related groups.

**Table 3:** Data on the 918 infections in relation to the presence or absence of neutropenia

	No neutropenia, *n* (%)	Neutropenia, *n* (%)	Not assessed, *n* (%)
Episodes of neutropenia, *n* (%)	274 (29.8)	30 (3.3)	614 (67)
Type of infection			
Bacterial	222 (81.0)	24 (80.0)	503 (81.9)
Viral	37 (13.5)	2 (6.7)	70 (11.4)
Fungal	10 (3.6)	2 (6.7)	32 (5.2)
Mixed microorganisms	5 (1.8)	2 (6.7)	9 (1.5)
Localization			
Respiratory system	90 (32.8)	9 (30.0)	146 (23.8)
Urogenital system	78 (28.5)	6 (20.0)	265 (43.2)
Bone, skin and soft tissues	56 (20.4)	6 (20.0)	131 (21.3)
Gastrointestinal system	13 (4.7)	2 (6.7)	25 (4.1)
Nervous system, multiorgan, or unspecified location*	37 (13.5)	7 (23.3)	47 (7.7)
Invasiveness**			
Noninvasive infections	100 (36.5)	17 (56.7)	207 (33.7)
Invasive infections	19 (6.9)	5 (16.7)	25 (4.1)
No microbiological analyses performed	155 (56.6)	8 (26.7)	382 (62.2)
Severity			
Infections with outpatient care	179 (65.3)	17 (56.7)	505 (82.2)
Infections requiring in-hospital care	77 (28.1)	9 (30.0)	93 (15.1)
Infections necessitating intensive care	14 (5.1)	3 (10.0)	9 (1.5)
Deaths due to infections	4 (1.5)	1 (3.3)	7 (1.1)

*Infections in the nervous system, multiple organ infections, and unspecified location of infections are reported together here due to low incidences

**Invasiveness is defined as all infections with positive blood or cerebrovascular fluid cultures

### Neutropenia combined with hypocomplementemia and/or lymphopenia

The proportion of neutropenic episodes that occurred in patients meeting the SLICC ‘low complement’ criterion was 21.3%, and there was no strong evidence that the odds of infection between the groups were different (OR = 2.1, 95% CI 0.24;24.15; POA = 75.9%). The proportions of neutropenic episodes that occurred or did not occur in patients with meeting the SLICC ‘lymphopenia’ criterion were similar, and the estimated odds of infection were also similar between the groups (OR = 1.04, 95% CI  0.26;4.36, POA = 52.2%). The characteristics of the infections with or without hypocomplementemia and/or lymphopenia are listed in Table 4.

**Table 4:** Data on the 918 infections in relation to hypocomplementemia and lymphopenia (fulfilled SLICC criteria), regardless of the presence of neutropenia

	Hypocomp-lementemia, *n* (%)	No hypocomp-lementemia,* n* (%)	Lymphopenia, *n* (%)	No lympho-penia, *n* (%)
	393 (42.8)	525 (57.2)	677 (73.7)	241 (26.3)
Type of infection				
Bacterial	323 (82.2)	426 (81.1)	554 (81.8)	195 (80.9)
Viral	52 (13.2)	57 (10.9)	81 (12.0)	28 (11.6)
Fungal	18 (4.6)	26 (5.0)	27 (4.0)	17 (7.1)
Mixed microorganisms	0 (0.0)	16 (3.0)	15 (2.2)	1 (0.4)
Localization				
Urogenital system	149 (37.9)	200 (38.1)	246 (36.3)	103 (42.7)
Respiratory system	121 (30.8)	124 (23.6)	189 (27.9)	56 (23.2)
Bone, skin and soft tissues	71 (18.1)	122 (23.2)	148 (21.9)	45 (18.7)
Gastrointestinal system	16 (4.1)	24 (4.6)	24 (3.5)	16 (6.6)
Nervous system, multiorgan, or unspecified location*	36 (9.2)	55 (10.5)	70 (10.3)	21 (8.7)
Invasiveness**				
Noninvasive infections	136 (34.6)	184 (35.0)	230 (34.0)	90 (37.3)
Invasive infections	22 (5.6)	27 (5.1)	37 (5.5)	12 (5.0)
No microbiological analyses performed	235 (59.8)	314 (59.8)	410 (60.6)	139 (57.7)
Severity				
Infections with outpatient care	312 (79.4)	389 (74.1)	497 (73.4)	204 (84.6)
Infections requiring in-hospital care	67 (17.0)	112 (21.3)	145 (21.4)	34 (14.1)
Infections necessitating intensive care	10 (2.5)	16 (3.0)	25 (3.7)	1 (0.4)
Deaths due to infections	4 (1.0)	8 (1.5)	10 (1.5)	2 (0.8)

*Infections in the nervous system, multiple organ infections, and unspecified location of infections are reported together here due to low incidences

**Invasiveness is defined as all infections with positive blood or cerebrovascular fluid cultures

### Neutropenia, lymphopenia, and hypocomplementemia in relation to the invasiveness and severity of infections

Table 5 shows the estimated conditional ORs describing the associations between the invasiveness and severity of infections and confirmed neutropenia, confirmed absence of neutropenia, or not controlled. As shown, the odds for an invasive infection were higher in subjects who had confirmed neutropenia versus those who did not. The odds of having a severe infection were also higher in those subjects who had confirmed neutropenia versus those who did not. The regression models were adjusted for variables that represented whether or not patients had ongoing or a history of hypocomplementemia or lymphopenia during the study period, and the interaction between the two items. The coefficients for these covariates indicated that patients who had at least one documented episode of hypocomplementemia were less likely to have invasive infections than patients who had had no documented episodes of hypocomplementemia, conditional on the other covariates in the model. Patients who had at least one documented episode of lymphopenia over the study period were more likely to have higher severity of infections compared to those who had no documented episodes of lymphopenia, conditional on the other covariates in the model. Finally, patients who had at least one documented episode of hypocomplementemia and lymphopenia were less likely to have higher severity infections compared to those who had no documented episodes of either.

**Table 5:** Comparisons of the invasiveness and severity of infections in relation to neutropenia, hypocomplementemia, and/or lymphopenia

	Median OR (95% CI)^a^	POA^b^
Invasive vs. non-invasive infections^c^		
Not assessed vs. no neutropenia	0.52 (0.20; 1.35)	91.4%
Neutropenia vs. no neutropenia	7.08 (1.11; 57.9)	98.1%
Hypocomplementemia vs. no hypocomplementemia	0.26 (0.03; 1.79)	91.3%
Lymphopenia vs. no lymphopenia	0.77 (0.19; 3.24)	64.6%
Hypocomplementemia and lymphopenia vs. No hypocomplementemia and no lymphopenia^d^	0.97 (0.24; 3.51)	51.9%
Higher-severity infections^e^		
Not assessed vs. no neutropenia	0.31 (0.2; 0.47)	> 99.9%
Neutropenia vs. no neutropenia	2.85 (0.91; 9.06)	96.3%
Hypocomplementemia vs. No hypocomplementemia	0.59 (0.17; 1.93)	80.4%
Lymphopenia vs. no lymphopenia	1.83 (0.83; 4.12)	93.3%
Hypocomplementemia combined with lymphopenia vs. No hypocomplementemia and no lymphopenia^e^	0.46 (0.23; 0.91)	98.7%

^a^The median of the marginal posterior probability distribution for the odds ratio (OR), with 95% compatibility intervals (CI) defined by the 2.5% and 97.5% percentiles of the posterior distribution

^b^The posterior probability of association (POA) in the direction of the median point estimate

^c^OR produced by logistic regression, modeling the odds of invasive versus non-invasive infections (infections for which microbiologic analyses were not performed were removed)

^d^Calculated by combining the estimates for the main covariates of hypocomplementemia and lymphopenia and the estimate of the interaction between the two items

^e^OR produced by ordinal regression, modeling the odds of higher-severity infections

### Pathogens

Notably, we identified a high number of infections, managed in the outpatient care setting, which had not been further investigated using microbiologic analyses. Altogether, any microbiological analysis (cultures of blood, urine, sputum or nasopharyngeal swab were most common) was ordered in 437/918 infections (47.6%), but 98 of these analyses fell out negative. Thus, support for infection by any microbiologic analysis (i.e., culture, serology, or viral detection) was available in 339/918 infections (36.9%). The pathogens responsible for infections in the 30 cases with neutropenia are detailed in Supplementary Table 1. Of note, in only 2/30 infections (6.7%) were the patients entirely off glucocorticoid treatment, and opportunistic infections with *Pneumocystis jirovecii* were seen in two individuals who were receiving high daily doses of prednisolone. Overall, the most frequently detected bacteria causing infections were pathogens affecting the respiratory tract, urinary tract, and skin. *Escherichia coli and Enterococcus faecalis, Streptococcus pneumoniae, Haemophilus influenzae, Klebsiella pneumoniae, Staphylococcus aureus,* and *Pseudomonas aeruginosa* were the predominant bacteria in the cultures of patients. Viral infections were caused by herpes simplex virus and varicella zoster virus, cytomegalovirus and/or Epstein–Barr virus, and SARS-CoV-2. Fungal infections were predominantly caused by *Candida* species at different locations in the body.

## Discussion

Herein, we investigated the prevalence of neutropenia and the associations with confirmed infections in well-characterized Swedish subjects with SLE who were residing in Östergötland County over a 14-year time period [30]. We show that 12% of the patients experienced at least one episode of (usually, mild) neutropenia over time. In general, infections were common among the study population, but only a minority of the infections were associated with neutropenia. Nevertheless, both the invasiveness and severity of the infections were significantly associated with neutropenia, whereas ongoing or a history of lymphopenia and/or hypocomplementemia did not affect the association to the same extent.

Although the *KLURING* cohort contains mainly patients of Caucasian ethnicity, our findings are similar to those recently published for the Toronto Lupus Cohort, in which isolated neutropenia was associated with Black race [36]. A systematic review from 2015 reported a slightly higher prevalence (range 20%‒40%) of neutropenia in patients with SLE [37]. It cannot be excluded that the divergent results are related to differences in SLE phenotypes, ethnicities, and/or the selection of overall more severely affected patients with more-severe SLE requiring more or higher doses of immunosuppressive agents [38]. In line with the observations made by others, we found that approximately two-thirds of all the episodes of neutropenia in SLE were mild (ANC < 1.5 × 10^9^/L ‒ ≥ 1.0 × 10^9^/L) [25]. Furthermore, we observed that neutropenia was usually accompanied by other hematologic abnormalities, such as anemia, leukopenia and lymphopenia, as well as the presence of anti-SSA antibodies (62%). The latter has also been observed by others, and Kurien et al*.* suggested that anti-SSA antibodies are directly involved in mediating neutropenia in patients with SLE [39].

The use of immunosuppressive agents is an obvious independent risk factor for neutropenia due to drug toxicity-induced medullary hypoplasia [25]. In addition, the use of rituximab in patients with SLE has been associated with late-onset neutropenia [26]. However, treatment with glucocorticoids (corresponding to a daily dose of 15 mg prednisone) may lead to a significantly increased number of neutrophils in the circulation [40]. In the present study, 62% of the patients used prednisone dosages ≤ 5 mg when the neutropenia was identified, and 37% used immunosuppressive agents. This indicates that most of the neutropenic episodes detected in this study are more likely part of the SLE pathogenesis per se than the side effects of immunosuppression, as mentioned previously [41]. Obviously, high doses of glucocorticoids have multiple effects on other blood cells; e.g., circulating lymphocyte levels may decrease, but could also be a result of SLE with different degree of severity [19]. NLR is frequently used as a prognostic marker in critically ill patients with infections [42]. In SLE, some studies have found elevated NLR useful to distinguish infections from flares whereas other claim that high NLR rather may be associated to active SLE, activation of the classical complement pathway, advanced organ damage, severe depression, and poor quality of life [43–46]. Our study did not observe any statistically significant difference in NLR between neutropenic patients with mild and severe infection.

Neutrophils play essential roles in host immune defenses, as they ingest, kill, and digest invading microorganisms. Interestingly, new data indicate that neutrophils may also play an important role in the resolution of inflammation [47]. However, this phenomenon has not yet been studied in detail in SLE. Although neutropenia is a far less common hematologic manifestation of SLE than lymphopenia, the overall interest in neutrophils in the context of autoimmunity, and particularly SLE, has grown significantly over the last decade. Impaired handling of apoptotic cells with exposure of modified self-antigens from dying cells, including NETs in a proinflammatory environment, affects immune tolerance with subsequent development of autoimmunity and deterioration of already established autoimmune disease [29, 48]. Exposure to the immune system of otherwise hidden molecules, such as DNA and nuclear constituents, logically represents a starting step for autoimmunity, and in SLE and antiphospholipid syndrome (APS), a functional neutrophil is important for preventing loss of tolerance via immune regulation and clearance [41, 49–51]. In support of this, NETs have been shown to contribute to inflammation and tissue damage in a number of other organs in patients with SLE, such as the brain, heart, and lung [52–54].

In the current study, approximately 80% of the infections observed were deemed to be bacterial, and they mainly affected the respiratory and urogenital tracts, skin, bone and soft tissues. Similar findings have previously been reported by the Spanish Rheumatology Society Lupus Registry regarding severe infections and bacteremia [13, 14]. Only a minority of the infections in the present cohort were severe and, thus the majority were managed with outpatient care regardless of neutropenia, probably reflecting effective health controls and access to the public healthcare system in this region of Sweden. Consequently, neutrophil counts were not assessed in conjunction with the majority of the infections. As a result, cultures and serologic tests for different causal microorganisms were not performed for > 50% of the infections, as they were managed as outpatients or were mild cases.

When the patients were divided based on ANC, to compare the infections, higher odds of severe infections were noted for patients with neutropenia, as compared with those patients who did not have neutropenia. In addition, the odds of developing invasive infections were also higher for subjects with confirmed neutropenia in our study. In agreement with a recent report, we did not find any evidence that lymphopenia aggravate the odds of infection [36]. A meta-analysis focusing on characteristics and risk factors of infection in SLE showed that after, adjusting for several factors, lymphopenia was a less important risk factor for infection whereas continuous complement consumption appeared to be worse [55].

There are some limitations to the present study that need to be considered when interpreting our findings. For this observational study, we did not include any control subjects with other diseases, and we did not have enough statistical power to evaluate any influence of immunosuppressive therapies. In addition, the relatively few neutropenic episodes in the dataset make it difficult to estimate the odds ratio with high precision and causes of neutropenia were not investigated (e.g., drug-induced neutropenia). Moreover, most of the cases included were outpatients for whom WBC differentiation tests had not been performed; thus, it is possible that some mild neutropenic episodes might have been missed. Nevertheless, the study is a monocenter cohort study with a well-controlled study population that was followed systematically over a 14-year period. Finally, it should be emphasized that the Swedish healthcare system is public, tax funded, and offers universal access, which constitute major strengths.

In conclusion, infections are common in patients with SLE and occur in both the presence and absence of neutropenia. Nevertheless, confirmed neutropenia co-appearing with infections seems to be associated with both the invasiveness and severity of infections, whereas a history of lymphopenia and/or hypocomplementemia did not seem to be as important in this context. Our results have implications for healthcare institutions in suggesting that they perform WBC differentiation testing as part of the risk stratification for patients with SLE who show signs of infection. Since infections still constitute a leading cause of mortality in SLE, neutropenia is relevant and important to consider in patients who have infections, to prevent severe illness and death.

### Supplementary Information

Below is the link to the electronic supplementary material.

Supplementary file1 (DOCX 22 KB)

## Data Availability

All data supporting the findings of this study are available within the paper and its Supplementary Information.
