# Supplementary material for: The prevalence of neutropenia and association with infections in patients with systemic lupus erythematosus: a Swedish single-center study conducted over 14 years
Source: Rheumatol Int. 2024 Mar 19;44(5):839–49. doi: 10.1007/s00296-024-05566-9 (PMC10980633; doi:10.1007/s00296-024-05566-9)
Supplement: Supplementary file 1 — Supplementary file1 (DOCX 22 KB) [file 296_2024_5566_MOESM1_ESM.docx]

**Supplementary Table 1:** Profiles of the 30 infections (15 individuals with SLE) that occurred in association with confirmed neutropenia.

| **Patient**  **number** | **Type of**  **neutropenia** | **Type of infection** | **Microbiologic**  **analyses** | **Prednisolone dose** (mg/day) | **Antimalarial**  **agent** | **Immunosuppressive**  **agent** | **Infection**  **severity*** | **Neutrophil-to-**  **lymphocyte ratio** |
| --- | --- | --- | --- | --- | --- | --- | --- | --- |
| 1 | Mild | Pneumonia | Negative | 7.5 | HQ | AZA | 1 | 0.52 |
| 2 | Moderate | Osteomyelitis | *E. coli* | 10 | 0 | 0 | 2 | 1.29 |
| 3 | Mild | Pneumonia | *Pneumocystis jirovecii* | 50 | 0 | 0 | 3 | 1.10 |
| 4 | Mild | Pneumonia | Not performed | 0 | 0 | Sirolimus | 1 | 1.27 |
| 5 | Moderate | Acute bronchitis | *Streptococcus pneumoniae* | 12.5 | HQ | 0 | 2 | 0.54 |
| 5 | Moderate | UTI | *Klebsiella pneumoniae* | 10 | 0 | 0 | 1 | 1.00 |
| 5 | Moderate | Pneumonia | Negative | 10 | 0 | Sirolimus | 2 | 0.40 |
| 5 | Moderate | Influenza + pneumonia | Influenza AH1N1 + *Pneumocystis jirovecii* | 25 | 0 | 0 | 2 | 1.20 |
| 5 | Moderate | Upper respiratory tract infection | Not performed | 12.5 | 0 | 0 | 1 | 0.83 |
| 5 | Severe | UTI | *Klebsiella pneumoniae* | 15 | 0 | Rituximab | 1 | 0.18 |
| 5 | Severe | UTI | *E. coli* | 10 | 0 | Cyclosporine | 1 | 0.20 |
| 5 | Severe | Pneumonia | *Haemophilus influenzae* | 12.5 | 0 | 0 | 2 | 0.38 |
| 5 | Severe | Pneumonia | Not performed | 12.5 | 0 | 0 | 1 | 0.38 |
| 5 | Severe | Abscess | *Staphylococcus aureus* | 15 | 0 | 0 | 1 | 0.11 |
| 5 | Severe | UTI | Coagulase-negative staphylococci | 12.5 | HQ | 0 | 1 | 0.15 |
| 5 | Severe | Abscess | *Staphylococcus aureus* | 10 | 0 | Cyclosporine | 1 | 0.17 |
| 5 | Mild | Erysipelas | Not performed | 10 | 0 | Cyclosporine | 1 | 1.00 |
| 5 | Mild | Influenza + pneumonitis + UTI | Influenza A virus + *E. coli* | 10 | 0 | Cyclosporine | 2 | 1.00 |
| 5 | Mild | UTI | Not performed | 10 | 0 | Cyclosporine | 1 | 1.00 |
| 6 | Mild | Herpes keratitis | Herpes simplex virus | 5 | 0 | Sirolimus | 1 | 1.50 |
| 7 | Mild | Oral candidiasis | Not performed | 50 | HQ | Rituximab | 1 | 2.50 |
| 8 | Agranulo-cytosis | Sepsis | Negative | 15 | 0 | MMF | 3 | 0.00 |
| 9 | Mild | UTI | *Pseudomonas aeruginosa* | 5 | 0 | MMF | 2 | 1.00 |
| 10 | Mild | Sepsis + necrotizing fasciitis | Hemolytic group G streptococci + *E. coli* | 0 | 0 | Rituximab | 4 | 1.57 |
| 11 | Mild | Lyme disease (skin) | Not performed | 10 | HQ | Dapsone | 1 | 1.30 |
| 12 | Mild | HIV infection | HIV-1 virus | 40 | HQ | 0 | 1 | 0.42 |
| 13 | Moderate | Urogenital candidiasis | Not performed | 10 | HQ | MTX | 1 | 0.50 |
| 14 | Moderate | Septic enteritis + oral candidiasis | Salmonella spp*. + Pseudomonas aeruginosa* | 30 | HQ | Rituximab | 2 | 0.32 |
| 15 | Mild | UTI + Pneumonia | *E. coli* + Ruminococcus spp. + Parvovirus 19 + CMV | 10 | HQ | Rituximab | 3 | 1.11 |
| 15 | Agranulo-cytosis | UTI + Neutropenic fever | *E. coli* | 10 | HQ | Rituximab | 2 | 0.11 |

*Severity of infections are graded as follows: 1 = outpatient; 2 = admitted cases; 3 = cases admitted to intensive care; 4 = death due to infection.

AZA, azathioprine; CMV, cytomegalovirus; *E. coli*, *Escherichia coli*; HIV, human immunodeficiency virus; HQ, hydroxychloroquine; MMF, mycophenolate mofetil; MTX, methotrexate; UTI , urinary tract infection.
